# Monitoring the Activation of the DNA Damage Response Pathway in a 3D Spheroid Model

**DOI:** 10.1371/journal.pone.0134411

**Published:** 2015-07-30

**Authors:** Odile Mondesert, Céline Frongia, Olivia Clayton, Marie-Laure Boizeau, Valérie Lobjois, Bernard Ducommun

**Affiliations:** 1 Université de Toulouse; ITAV-USR3505, F-31106 Toulouse, France; 2 CNRS; ITAV-USR3505, F-31106 Toulouse, France; 3 CHU de Toulouse; F-31059 Toulouse, France; Institut de Génétique et Développement de Rennes, FRANCE

## Abstract

Monitoring the DNA-Damage Response (DDR) activated pathway in multicellular tumor spheroid models is an important challenge as these 3D models have demonstrated their major relevance in pharmacological evaluation. Herein we present DDR-Act-FP, a fluorescent biosensor that allows detection of DDR activation through monitoring of the p21 promoter p53-dependent activation. We show that cells expressing the DDR-Act-FP biosensor efficiently report activation of the DDR pathway after DNA damage and its pharmacological manipulation using ATM kinase inhibitors. We also report the successful use of this assay to screen a small compound library in order to identify activators of the DDR response. Finally, using multicellular spheroids expressing the DDR-Act-FP we demonstrate that DDR activation and its pharmacological manipulation with inhibitory and activatory compounds can be efficiently monitored in live 3D spheroid model. This study paves the way for the development of innovative screening and preclinical evaluation assays.

## Introduction

The DNA-Damage Response (DDR) pathway activated in response to DNA injury has been the subject of major investigation, leading to the identification of sensors, transducers and effectors which ensure the transduction of information and the activation of the appropriate responses, including DNA repair machinery, proliferation arrest and potentially cell death [1]. The role of major actors of this pathway such as the p53 tumor suppressor protein and its transcriptional target p21, a well-known inhibitor of CDK-Cyclin complexes whose accumulation is responsible for cell cycle arrest, is largely documented [2]. Indeed, in response to DNA damage, activated checkpoint kinases phosphorylate p53, which in turn is stabilized and escapes from rapid mdm2 ubiquitin ligase-dependent degradation by the proteasome [3]. Consequently, p53 accumulates and activates the transcription of multiple targets including p21 and GADD45.

Live monitoring of DDR activation in multicellular structures and in tissues remains poorly investigated. Indeed, most studies rely on immunohistochemistry performed on fixed tissue sections stained with antibodies against DNA damage foci (phosphorylated form of **γ**H2AX), activated kinases (phosphorylated epitopes on ATM, CHKs), or against p53 or p21 to assess their relative levels [4] [5] [6] [7]. It is therefore essential to develop new experimental approaches and new biological tools to allow the exploration of DDR activation within live tissues, thus aiming to improve our understanding of the involved mechanism in a 3D context and to develop new assays for pharmacological evaluation.

The 3D multicellular spheroid is the ideal model to setup a new experimental strategy that fulfills these needs. Its size and progressive regionalization associated with a proliferation gradient installed during its growth makes the spheroid a genuine model mimicking the organization found in tissues or in tumoral micro domains. It is thus now widely accepted that spheroids accurately reproduce the 3D architecture of solid tumors, bridging the gap between monolayer cultured cells and animal models[8]. Consequently, their interest as models to evaluate new anti-cancer strategies is increasingly recognized [9].

In the study presented here, we report the engineering of the DDR-Act-FP biosensor and its pharmacological validation in a cancer cell line cultivated in 2D. We then present the use of this reporter expressing cell line to screen a small compound library to identify DDR response modulators. Finally, we use 3D spheroids to demonstrate the major interest of DDR-Act-FP reporter use to automatically quantify DDR activation kinetics upon exposure to DNA damage and to monitor its pharmacological manipulation.

## Materials and Methods

### Cell line engineering

A 2.3 kb cDNA fragment encompassing the p21 promoter region of the p21/CIP1 cell cycle inhibitor was cut out from the WWP-Luc cDNA (Addgene16451). The cDNA encoding the mRFP fluorescent protein (a generous gift from R. Tsien laboratory) was cloned downstream from the p21 promoter cDNA. This construct was then transferred to the pTRIP lentiviral shuttle vector previously deleted from the CMV promoter. The resulting plasmid (pTRIPΔCMV-Act-mRFP) was used to produce lentiviral particles in 293FT embryonic kidney cells (Life Technologies) after calcium chloride tri-transfection together with pGag/pol and pVSV-G plasmids (provided by Vectorology platform, INSERM U1037). 7 hours post transfection, DMEM+Glutamax (Gibco by Life Technologies) supplied with 10% FCS was washed out and replaced with serum free OPTIMEM+Glutamax (Gibco by Life Technologies). Lentiviral particles were harvested 48 hours later and titer was quantified by flow cytometry (BD Accuri C6) on HT1080 cells (ATCC), transduced with serial dilutions of lentivirus. HCT116 p53 proficient colorectal cancer cells (obtained from ATCC) were then transduced at a MOI of 6 in the presence of 4μg/ml protamine sulfate in OPTIMEM+Glutamax. Medium was replaced 7 hours later with DMEM+Glutamax with 10% FCS. We thus generated a stable HCT116 DDR-Act-FP expressing cell line. Single cell clonal isolation was performed on 96 well plates. Wells containing single clones were trypsinized and cells seeded in 96 well flat bottom plates for 3 days, treated for 24 hours with 20μM Nutlin-3, 0.5μM Etoposide, or 10μM Etoposide and then fixed and stained with DAPI before being scanned for total fluorescence intensity (CircSpotTotalIntensity) as described below. Clones showing a fluorescence enhancement upon drug treatments were then submitted to concentrations of Etoposide ranging from 0.5 to 20μM. Ultimately, selection was determined upon response to ATM inhibitors KU-55933 and CP-466722 (Selleckchem).

### Cell culture and spheroids production

HCT116-DDR-Act-FP cells were cultured in DMEM+Glutamax containing 10% FCS with penicillin/streptomycin in a humidified atmosphere of 5% CO2 at 37°C. Spheroids were prepared exactly as described in previous publications [6]. Briefly, 1000 cells per well were distributed in poly-HEMA-coated 96-round bottom well plates. Plates were centrifuged (300 g for 6 min) and then placed in a humidified atmosphere of 5% CO2 at 37°C. Spheroids were treated after 3 days for the duration and at the concentration of drugs indicated for each experiment. At the initial stage (approx. 300μm in diameter) no regionalization of proliferation is observed and the spheroid cell population is actively proliferating. After 6 days (*i*.*e*. 3 days of treatment), control spheroids are approximatively 450–500μm in diameter, regionalization starts to occur with cells entering quiescent stage in the innest region. However, at that stage, no necrotic core is observed.

### Pharmacological treatment and LOPAC library screening

The ATM inhibitors KU-55933 (S1092) and CP-466722 (S2245) were obtained from Selleckchem. LOPAC-1280 library, Nutlin-3 and Etoposide were purchased from Sigma-Aldrich.

Screening of the LOPAC small compound library was performed as follows: 10 000 cells per well were seeded in 96-well flat bottom plates (Corning CellBind CLS3340-50EA), 24 hours later cells were treated with drugs for 24 hours at a final concentration of 5μM in 0.5% DMSO in the presence of 0.5μM Etoposide, or with 0.5μM Etoposide in drug-free DMSO (negative control) or with DMSO only. Cells were then fixed for 10 minutes in 10% neutral buffered formalin solution (Sigma HT5012), nuclei stained for 10 minutes with DAPI (1μg/ml in PBS) and stored at 4°C until scanned (Thermo Cellomics Compartmental Analysis V4). Acquisitions were made with a 20x objective, both with XF93-Hoechst filter, 0.05sec exposure (DAPI) and XF93-TRITC filter, 0.5sec (RFP).

### Monitoring DDR-Act-FP in 2D cultured cells

The fluorescence of DDR-Act-FP was quantified using the Cellomics Scan software. 500 cells per well were scored and nucleus dye used to identify primary objects (nuclei) in channel 1, thus defining the primary mask. The Circ, 10 pixels larger than the primary object, was used to define the cellular area of interest. Circspots are punctuate objects within the Circ area identified over a given intensity threshold, which in the study is 50. We therefore quantified fluorescence intensity using CircSpotTotalIntensity in channel 2.

### Monitoring DDR-Act-FP in 3D spheroid

Acquisitions were made with a 5x objective, both with XF53-Brightfield, 0.0002sec exposure and XF53-TexasRed filter, 0.002sec (RFP). Images of spheroids were acquired using the Compartmental Analysis V4 tool. Nine fields were acquired for each well in the case the spheroid should be off-center and therefore incorrectly segmented. Images were segmented on the bright-field channel (channel 1) with an ObjectAvgInten maximum of 1500 and a ObjectArea minimum of 15000. Segmentation was then replicated to channel (RPF) where fluorescence was quantified. When off-center in a field and therefore ineligible for segmentation, a new output field was added to a well acquisition using the Montage Tool associated with Cellomics Scan software. Relative fluorescence level was read by extracting MeanCircAverageIntensity parameter in the fluorescent channel 2. After visual verification of image quality, the mean response of 6 spheroids per condition was normalized by dividing by the mean response of 6 untreated spheroids, giving a response factor of 1 (identical to untreated response) or higher.

### Western blot

Western blots were performed using whole cell extracts obtained by lysing the cells in NuPAGE LDS Sample Buffer (Novex by Life Technologies) in presence of NuPAGE Sample Reducing Agent. Proteins were separated using NuPAGE 4–12% Bis-Tris gradient precasted gels (Novex), transferred to Nitrocellulose and immunostained using antibodies against p53 (DO-1 SC126), **γ**H2AX (05–636 Cell Signaling) and actin (MAB1501 Chemicon).

## Results

### DDR-Act-FP: A Fluorescent reporter for the detection of DNA Damage Response pathway activation

**Fig 1:**
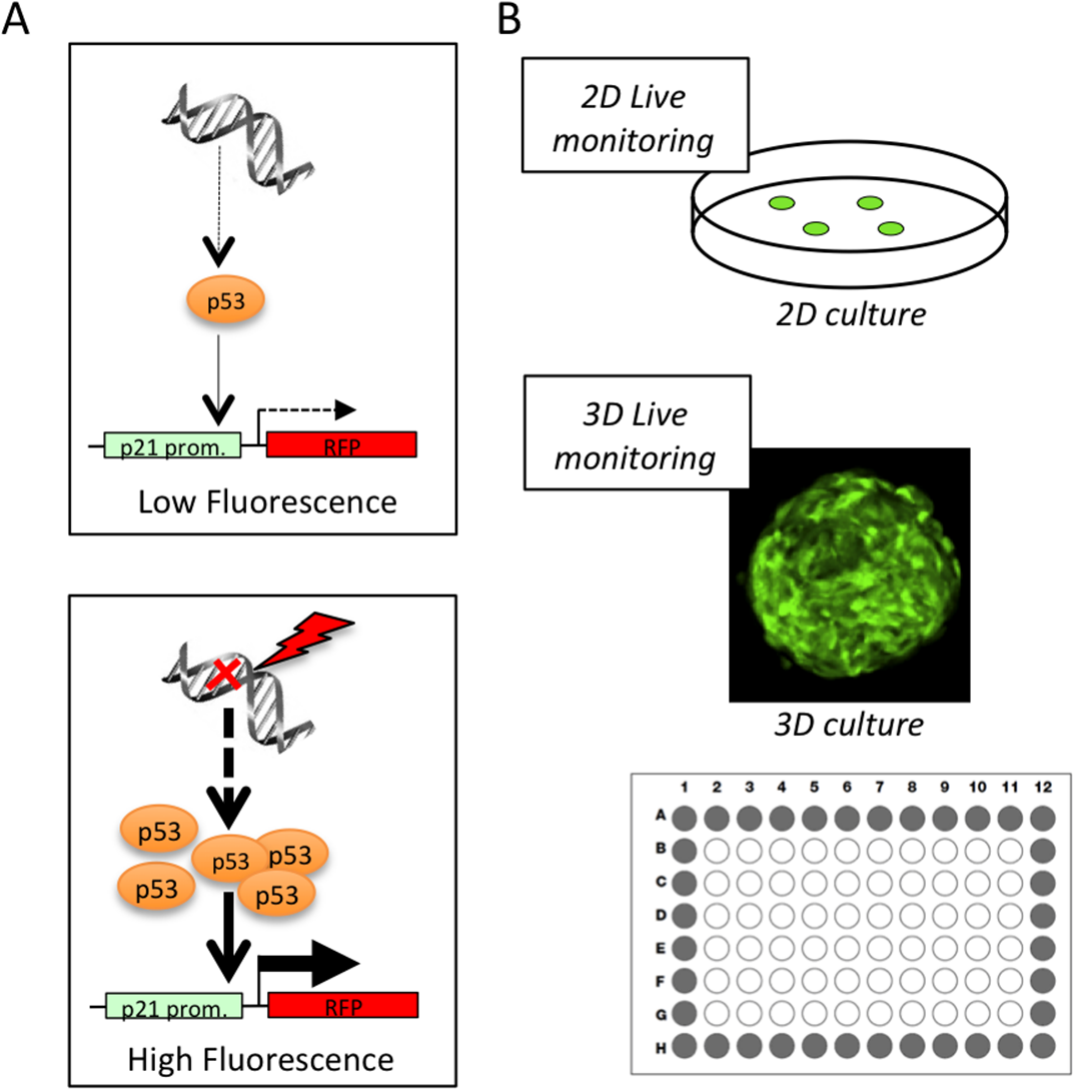
A Fluorescent assay to monitor DDR pathway activation. (A) Schematic representation of the principle of the DDR-Act-FP reporter. Upon DNA-damage dependent accumulation of p53, transcriptional activation of the p21 promoter leads to the expression of the fluorescent protein (FP). (B) Principle of the assay. A cell line stably expressing the DDR-Act-FP reporter is used to produce 3D spheroids. DDR pathway activation can be monitored globally on 2D monolayer cultured cells and on 3D spheroids grown in 96 well plates using a High Content Screening approach.

A reporter of the DNA Damage Response (DDR) pathway activation was constructed based on the schematic summary presented in Fig 1. The coding sequence of the mRFP fluorescent protein was cloned under the control of the human p21/CIP1 whole promoter sequence [10] with the aim of monitoring the p53-dependent transcriptional regulation of p21 expression. Thus upon activation of the DDR pathway, increase in p53 levels should result in a sustained expression of mRFP and enhanced fluorescence levels. This reporter system was designed to allow monitoring of the DDR pathway activation in 2D cultured cells and in 3D spheroid models using a high content imaging approach (Fig 1B).

**Fig 2:**
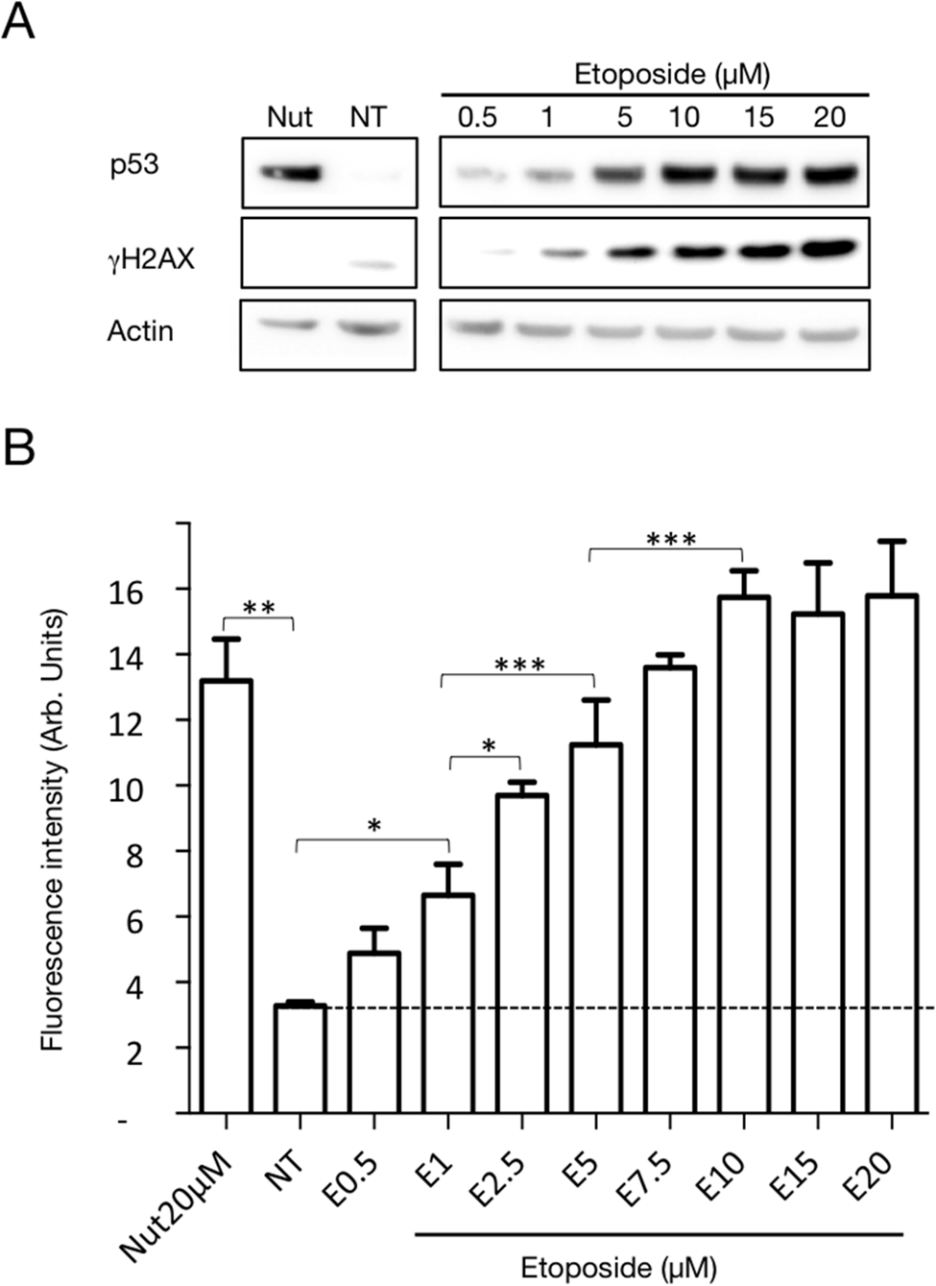
Characterization of the cell line expressing the DDR-Act-FP reporter. HCT116 cells stably expressing the DDR-Act reporter were left untreated (NT) or treated with Nutlin-3 (Nut 20 μM), or with Etoposide concentrations from 0.5 to 20 μM (E0.5 to E20) for 24 hours. (A) Western blot analysis of p53, **γ**H2AX and actin level. (B) Bar chart represents the average fluorescence intensity+/-SD monitored after 24h. 1500 cells from 3 independent wells were analyzed for each condition using the Cellomics scan software. *:P<0.05; **:P<0.01; ***:P<0.005 (One-way ANOVA, Prism).

As described in the methods section, lentiviral transduction was used to produce an HCT116 p53 proficient cell line stably and homogeneously expressing the DDR-Act-FP reporter. In order to characterize it, this modified cell line was treated for 24 hours with either 20μM Nutlin-3 or increasing concentrations of the DNA damaging agent Etoposide. As expected, treatment with Nutlin-3, a compound that prevents interaction between p53 and Mdm2 [11] [12] [13], led to an increase in p53 level detected by western blot analysis (Fig 2A) that, in turn, resulted in a major increase in DDR-Act-FP dependent fluorescence (Fig 2B). Similarly, treatment with the DNA-topoisomerase II inhibitor Etoposide (0.5 to 20 μM) resulted in a dose-dependent increase in p53 levels that paralleled DNA damage detected with **γ**H2AX labelling and consequently a proportional increase in fluorescence intensity associated with the reporter expression (Fig 2A and 2B).

### DDR-Act-FP: A sensor for pharmacological manipulation of the DDR pathway

**Fig 3:**
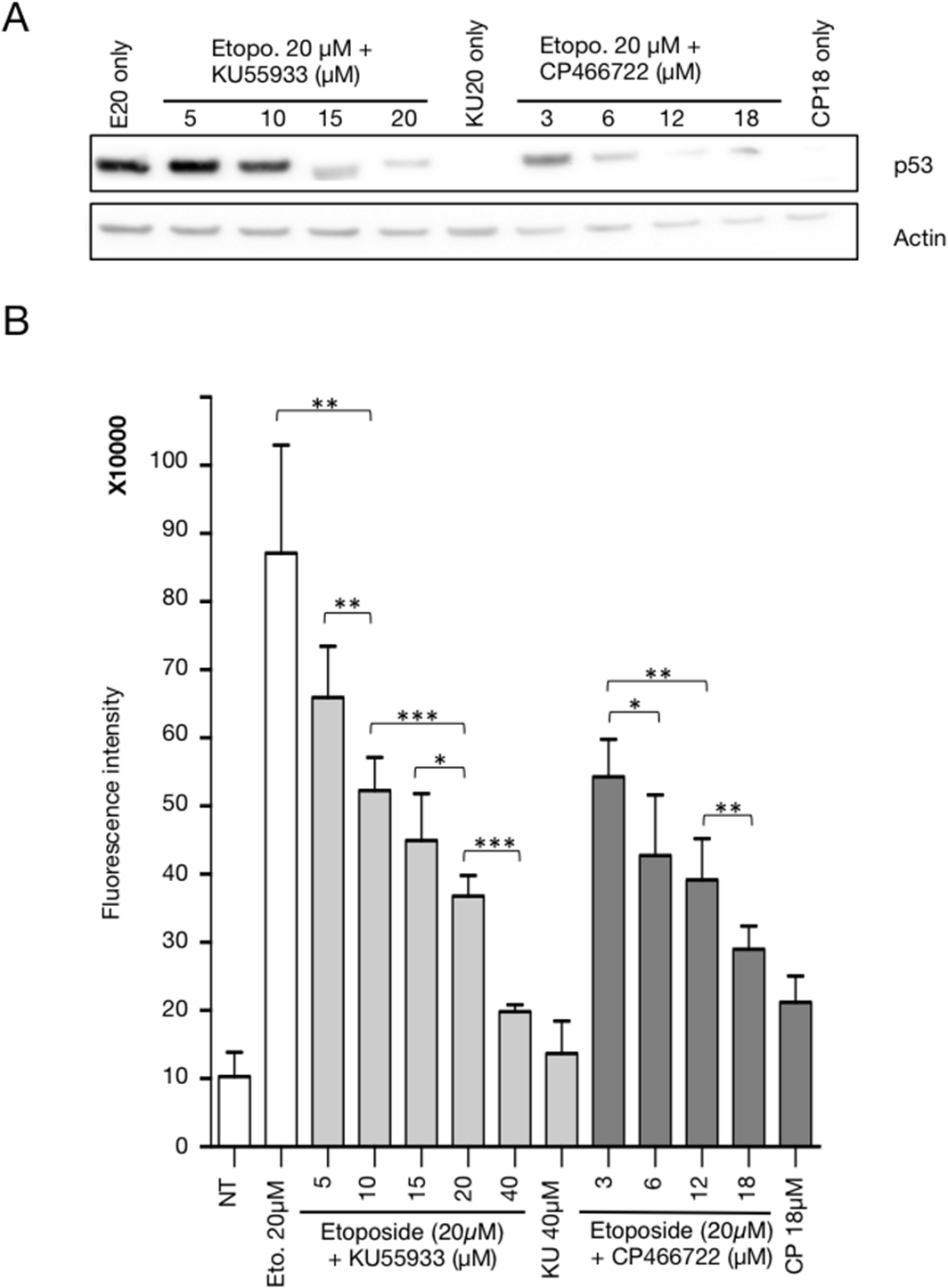
Pharmacological manipulation of the DDR-Act-FP reporter using ATM kinase inhibitors. HCT116 cells expressing the DDR-Act reporter were treated with Etoposide 20 μM (E20) for 24 hours, in the absence or in the presence of KU-55933 or CP-466722 at the indicated concentration. (A) Western blot analysis of p53 and actin as control. (B) Fluorescence level was monitored after 24h. Bar graph represents the average fluorescence intensity+/-SD from 6 samples (500 cells analysed/sample with Cellomics scan software) for each condition. *:P<0.05; **:P<0.01; ***:P<0.005 (Unpaired t-test, Prism).

Manipulation of the DDR pathway is a promising avenue for new therapeutic strategies [14] [15]. Consequently, as a prerequisite for the use of the DDR-Act-FP sensor in a 3D spheroid model for the validation of new compounds, we examined whether this reporter could accurately monitor pharmacological manipulation of the DDR pathway. To address this issue, we used KU-55933 and CP-466722, two inhibitors of the ATM kinase [16] [17] [18] and examined whether the DDR-Act-FP sensor could report the inhibitory effect of these two compounds on the DNA-damage dependent activation of the DDR pathway. The HCT116 cell line expressing the DDR-Act-FP sensor was treated with 20μM Etoposide alone or together with increasing concentrations of KU-55933 and CP-466722. As shown in Fig 3A, this treatment resulted in a dose-dependent inhibition of the increase in p53 protein levels as compared to the control induction observed in the presence of Etoposide alone. This observation is in agreement with the fact that, upon inhibition of the ATM catalytic activity, downstream activation of the checkpoint kinase 2 required to phosphorylate and stabilize p53, is restricted [19,20]. As expected, the lack of p53 accumulation was accompanied by a substantial and dose-dependent decrease in DDR-Act-FP fluorescence levels observed in the presence of 20μM Etoposide (Fig 3B). A similar result was observed with both ATM inhibitors.

Altogether the results presented in Figs 2 and 3 demonstrate that the cell line expressing the DDR-Act-FP reporter can be used to monitor DDR activation and to explore the pharmacological manipulation of the DNA damage response pathway.

### DDR-Act-FP: A valuable tool to screen small compound libraries

In order to further validate the potential of this reporter assay as a tool to identify pharmacological modulators of the DDR pathway, we set up the following pilot screen. HCT116 DDR-Act-FP cells were exposed to 0.5 μM Etoposide, a concentration that we have shown to result in a modest fluorescence increase over the background signal (see Fig 2B). We selected this specific experimental situation to screen the LOPAC commercial library (1,280 compounds) searching for small compounds further activating the DDR pathway and therefore enhancing the monitored fluorescence signal. The quantification of the monitored signal was performed with reference to a positive control that we set up as the fluorescence intensity measured when cells are treated with 10μM Etoposide (Table 1). As a second positive control, Nutlin-3 was also introduced into the assay and indeed was detected as the top hit that strongly enhanced the DDR-Act-FP reporter signal (112% of the Etoposide positive control). As expected, a significant number of known genotoxic compounds were identified in this blind screen for their ability to significantly increase the DDR-Act-FP fluorescence level. The top-score identified compounds are listed in Table 1. They represent 50% of the known genotoxic compounds that are included in the LOPAC library. This result confirms that the DDR-Act-FP reporter can efficiently be used as a biosensor to identify DNA damaging agents and for the detection of DDR pathway activation in response to genotoxic exposure.

**Table 1:** Known genotoxic compounds identified with the DDR-Act-Fp reporter system amongst 1,280 pharmacologically compounds of the LOPAC library. Results are expressed as percentage of the fluorescence intensity measured in cells treated with 10μM Etoposide. ID number, compound name and putative mode of action are indicated.

*Fluorescence (% of pos*. *control)*	*ID*	*Compound name*	*Mode of action*
100	Pos. control	Etoposide	Topoisomerase II inhibitor
112	-	Nutlin-3	Mdm2-p53 interaction inhibitor
110	M6545	Mitoxantrone	DNA synthesis inhibitor
108	I1656	Idarubicin	Antineoplastic
104	A9809	Amsacrine	Topoisomerase II inhibitor
91	E1383	Etoposide	Topoisomerase II inhibitor
83	T2705	Topotecan	Topoisomerase I inhibitor
82	A8598	Ancitabine hydrochloride	Antineoplastic
80	C6645	Cytosine-1-ß-arabinofuranoside	DNA synthesis inhibitor
78	A6770	Methotrexate hydrate	Folic acid antagonist
76	A1784	Aminopterin	Dihydrofolate reductase inhibitor
73	G6423	Gemcitabine	Antineoplastic
64	F6627	5-Fluorouracil	Thymidilate synthetase inhibitor
63	F8791	5-Fluoro-5’-deoxyuridine	DNA synthesis inhibitor
59	E3380	Ellipticine	Topoisomerase II inhibitor
58	C9510	Pyrocatechol	Carcinogen

These data demonstrate that the HCT116 cell line expressing the DDR-Act-FP reporter fulfills the expected needs and can therefore be used to produce spheroids with the aim of exploring and manipulating the DNA damage response pathway in 3D models.

### DDR-Act-FP: A sensor for the dynamic monitoring and modulation of DDR activation in live 3D spheroids

**Fig 4:**
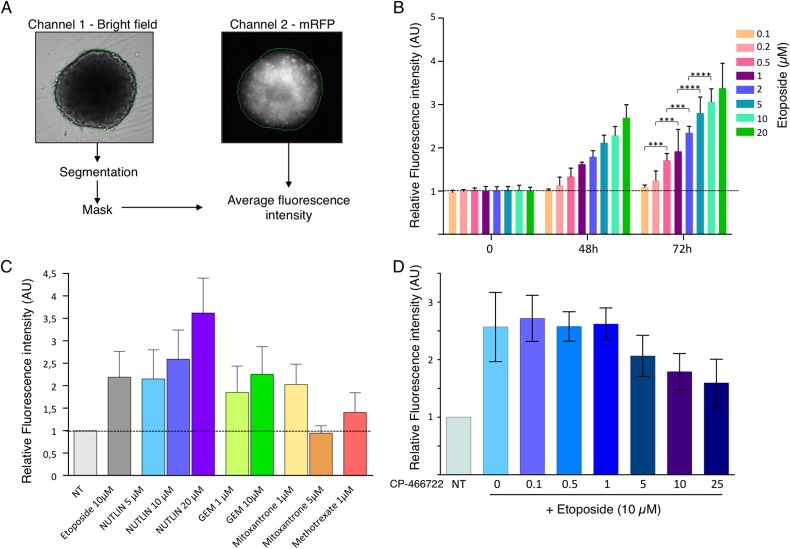
Use of DDR-Act-FP for the dynamic monitoring of DDR activation in live 3D spheroids. Spheroids were prepared with HCT116 cells expressing the DDR-Act reporter in 96 well plates. Relative fluorescence intensity to the untreated control was monitored on live spheroids at the indicated time and in the presence of the indicated compounds. The results are the average of determination on 6 spheroids for each experimental condition. (A) Image acquisition were automatically performed on an ArrayScan (Cellomics) using the Thermo Cellomics Compartimental Analysis V4 software. The bright field channel was used to perform segmentation and to create a mask used to extract the mRFP average fluorescence intensity from the second channel. A spheroid treated for 24 hours with 5μM etoposide was used in this illustration. (B) Bar graph shows the mean relative fluorescence intensity+SD after 48h and 72h of growth in the presence of the indicated concentrations of Etoposide (3 to 5 independent experiments, with 6 samples per condition). ***: P<0.005, ****: P<0.001. (C) Mean relative fluorescence intensity+SD after 24h in the presence of the indicated compounds. (D) Mean relative fluorescence intensity+SD after treatment for 24 hours with increasing concentration of the ATM inhibitor CP-466722 prior to incubation in the presence of 10μM Etoposide for 48 hours (3 independent experiments with 6 samples per condition for each).

The HCT116 DDR-Act-FP cell line was used to produce multicellular tumor spheroids in 96-well plates (as described in the methods section) thus allowing automated monitoring of multiple parameters including DDR-Act-FP-dependent fluorescence in live spheroids. Image acquisitions were performed on an ArrayScan (Cellomics) setup and analyses were carried out using the Thermo Cellomics Compartimental Analysis V4 software. Spheroids were segmented using the bright field channel, allowing extraction of area and determination of relative fluorescence intensity on the mRFP channel (Fig 4A).

This protocol was first used to evaluate the dynamic effects of 3D spheroid treatment with Etoposide concentrations ranging from 0.1 to 20 μM after 24 and 48 hours. As presented in Fig 4B, incubation of spheroids (6 for each condition per experiment in 96-well plates) in the presence of the DNA-damaging agent resulted in an increase in fluorescence intensity. Furthermore, both concentration-dependent and time-dependent effects on the fluorescence increase were observed (Fig 4B).

We next intended to validate this 3D model and experimental protocol using a panel of DDR pathway activators or modulating agents. To this aim, spheroids were treated for 24 hours with Nutlin-3 (5, 10 and 20μM) to stabilize p53, or with DNA damaging agents such as Etoposide (10μM), Gemcitabine (1 and 10μM), Mitoxantrone (1 and 5 μM) or Methotrexate (1μM). Spheroid fluorescence variation was monitored as above described. As shown in Fig 4C, activation of the DDR-pathway revealed by a significant increase in fluorescence was observed with most compounds. However, it is noteworthy that in several cases toxicity of the genotoxic compounds (i.e. Mitoxantrone at 5μM) led to major inhibition of cell proliferation and to cell death thus preventing further increase in fluorescence.

In light of what was reported in 2D cell culture, we then examined whether pharmacological manipulation of the DDR pathway activation could be monitored in live 3D spheroids. To this aim, the ATM inhibitor CP-466722 was used in the same ArrayScan setup as the previous experiment. Spheroids were treated for 24 hours with increasing concentrations of CP-466722 prior to their 48 hours incubation in the presence of 10μM Etoposide. As shown in Fig 4D, a dose-dependent inhibition of the DDR activation-dependent fluorescence signal was observed.

Altogether, these data validate the use of the HCT116 DDR-Act-FP 3D spheroid model to globally monitor activation of the DDR pathway, hence to detect the presence of genotoxic agents or to assess the effect of drugs interfering with the DDR pathway.

## Discussion

The aim of this work was to develop a fluorescent biosensor for reporting the activation of the DDR pathway in 3D multicellular tumor spheroids. The monitoring of DDR activation in cultured cells grown as monolayer is well described and the cascade of molecular events induced by DNA injury has been already documented using various type of reporters, including ATM kinase activity biosensors and relocalization of 53BP1 and XRRC1 [21] [22] [23]. It has for example been shown that foci formation of a 53BP1-GFP fusion protein can be used as live-cell imaging marker of UV-induced DDR activation in cells grown in monolayer culture and in 3D culture of superficial implanted tumors [24–26]. However, from a technical viewpoint none of these approaches can be translated to high throughput monitoring of DDR activation in whole large 3D spheroid. We previously reported the use of SPIM light sheet microscopy for live imaging of large spheroids (above 400μm in diameter) [27,28]. This new microscopy approach enables imaging cells deep inside spheroids and enables the detection of 53BP1-GFP foci in the nucleus of DNA-damaged cells (unpublished data). However, only a very limited number of live spheroids can be imaged at once [29]. To address this limitation, implementation of high throughput capability on SPIM light sheet imaging has been proposed, but it is still currently at the stage of instrumental development and not yet available for screening application.

Thus, the classical approach to assess DDR activation in 3D spheroid is based on immunohistochemistry and relies on the use of antibodies that detect specific events such phosphorylation of H2AX or ATM, or the accumulation of p53. This approach is validated and robust, but is not applicable to large scale screening strategy. Furthermore, immunohistochemistry requires fixation of the sample, thus preventing the possibility to perform monitoring of the response over time as shown in Fig 4B. Thus, to reach our goal, we constructed a reporter system based on the p53-dependent activation of the p21 promoter to express the mRFP fluorescent protein. This biosensor, cloned in the HCT116 cell line, was shown to report the activation of the DDR pathway upon treatment with genotoxic compounds such as Etoposide. This result was obtained with cells cultured as monolayer, but similarly with live whole 3D spheroids where we observed a dose-dependent and time-dependent increase in fluorescence levels (S1 Fig). It has been reported that p21 expression in spheroid is associated with proliferating cells and fluctuates overtime [30]. However, we have previously shown that in untreated HCT116 spheroids p21 expression is very limited and not associated to proliferating cells [7]. Hence, in the present study, we observed a limited background expression of the DDR-Act-FP reported that does not affect monitoring and quantification of global spheroid fluorescence.

Using this biosensor expressing cell line, we were able to perform a pilot screen using the LOPAC small molecule library that allowed us to demonstrate our ability to detect a large part of the compounds that are tagged as genotoxic in this commercial collection.

Manipulation of the DDR pathway is an attractive pharmacological approach that can be evaluated with the DDR-Act-FP promoter we have developed. As proof of concept, we report here that both in 2D culture and in 3D spheroids, the use of ATM inhibitors results in the inhibition of DDR activation.

Altogether, our results illustrate the potential offered by DDR-Act-FP expressing cell lines to evaluate known compounds in preclinical assays but also to perform original and innovative screening for new DDR modulators.

## Supporting Information

S1 Fig Schematic view of the data obtained in assays performed on 2D cells and on 3D spheroids to study the response to increasing concentration of Etoposide. (A) 2D cells are grown in 96-wells plates. Images are acquired and processed on an automated high throuput microscopy platform. Representative microscopy fields of channel 1 (DAPI) and channel 2 (mRFP fluorescence of DDR-Act-FP) are shown. Quantification of these data is presented in Fig 2B. (B) 3D spheroids are produced and grown in low attachment 96-wells plates. Images are acquired and processed on an automated high throuput microscopy platform. Representative images of spheroids in channel 1 (bright field) and channel 2 (mRFP fluorescence of DDR-Act-FP) are shown. Quantification of these data is presented in Fig 4B.
(TIF)
