# Supplementary figures and images for: Monitoring the Activation of the DNA Damage Response Pathway in a 3D Spheroid Model
Source: PLoS One. 2015 Jul 30;10(7):e0134411. doi: 10.1371/journal.pone.0134411 (PMC4520595; doi:10.1371/journal.pone.0134411)

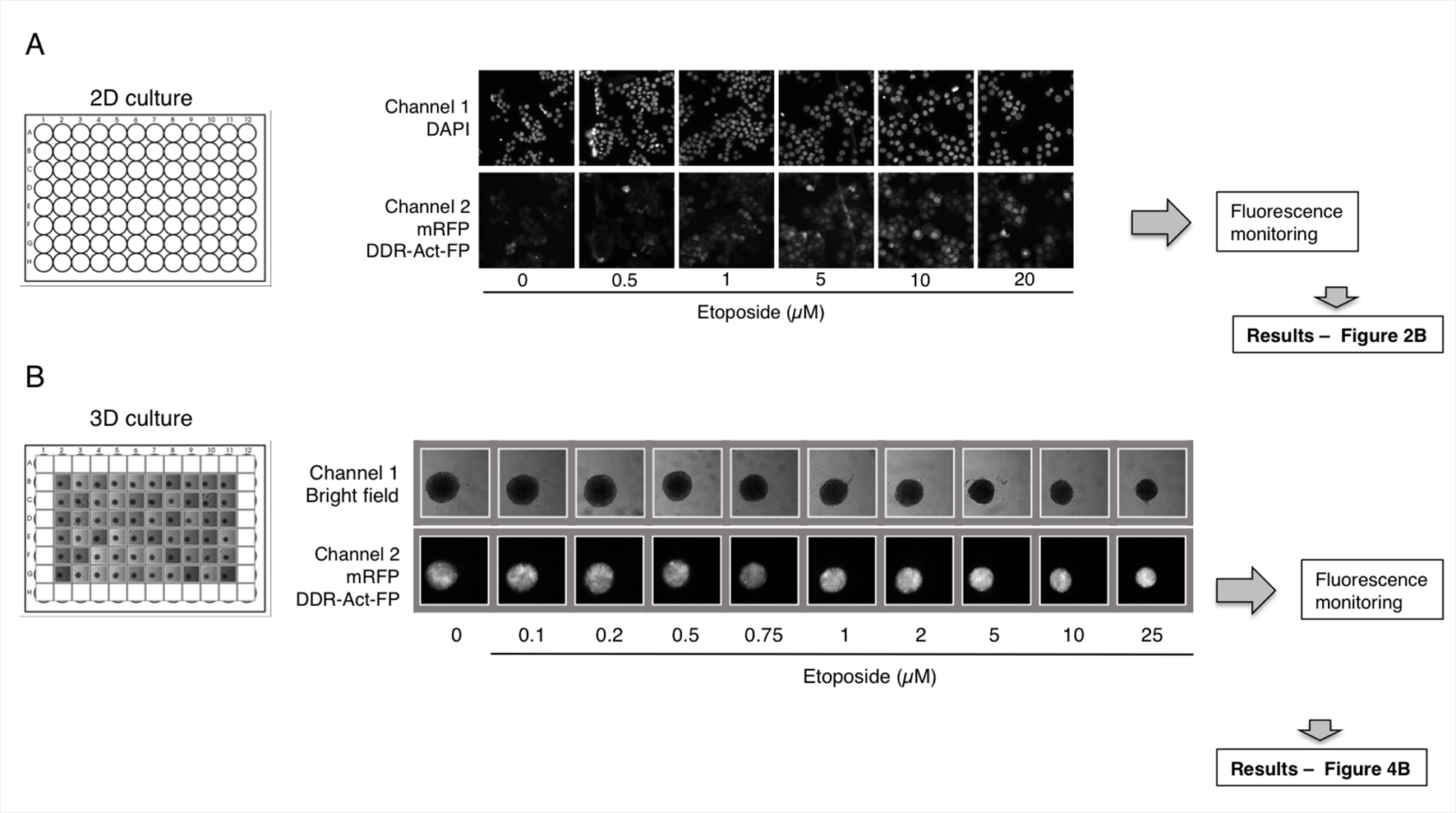

Supplement: S1 Fig — (A) 2D cells are grown in 96-wells plates. Images are acquired and processed on an automated high throuput microscopy platform. Representative microscopy fields of channel 1 (DAPI) and channel 2 (mRFP fluorescence of DDR-Act-FP) are shown. Quantification of these data is presented in Fig 2B. (B) 3D spheroids are produced and grown in low attachment 96-wells plates. Images are acquired and processed on an automated high throuput microscopy platform. Representative images of spheroids in channel 1 (bright field) and channel 2 (mRFP fluorescence of DDR-Act-FP) are shown. Quantification of these data is presented in Fig 4B. (TIF) [file pone.0134411.s001.tif]
